# Effect of Activated Siliceous Wastes Incorporated as Mineral Admixtures on the Rheological Properties of Cement Paste: Insights into Their Physicochemical Interactions in Suspension

**DOI:** 10.3390/ma17153781

**Published:** 2024-08-01

**Authors:** Linyun Shi, Jingzhong Kuang, Tingsheng Qiu

**Affiliations:** 1 Jiangxi Provincial Key Laboratory of Low-Carbon Processing and Utilization of Strategic Metal Mineral Resources, School of Resources and Environmental Engineering, Jiangxi University of Science and Technology, Ganzhou 341000, China; shilinyun1@163.com (L.S.); qiutingsheng@163.com (T.Q.); 2 Jiangxi Building Materials Research and Design Institute Co., Ltd., Nanchang 330001, China

**Keywords:** silicate mineral, fluidity, hydrophilia, cement paste, PCE, waste utilization

## Abstract

Mechanical grinding is a common method used to enhance the pozzolanic activity of tailings, and these activated tailings can be used as supplementary cementitious materials in cement production. However, the addition of activated tailings usually reduces the workability of cement paste, and the mechanism of influence of different minerals in tailings on workability varies. In this study, three kinds of principal silicate minerals in tailings—quartz, feldspar, and mica—were mechanically activated. The influence of these activated minerals on the rheological properties of cement paste were studied in the absence or presence of PCE (polycarboxylate ether) superplasticizers, and the influence mechanism was investigated using rheology, TOC, contact angles, zeta potential, XPS, ICP-OES, and XRD. The results showed that quartz has the highest fluidity, and mica has the lowest. An increase in hydrophilicity decreased the flowability of the blended cement paste. The increase in the metal cation dissolution rate was the main reason for the decrease in the fluidity of PCE-blended cement pastes. The knowledge gained provides a valuable reference for the utilization of activated tailings in cement production.

## 1. Introduction

Billions of tons of mine tailings are produced worldwide every year through mineral processing as a result of the increasing demand for minerals and metals [1]. Due to their low activity and fine particle size, high-value utilization is limited. The utilization of mine tailings as supplementary cementitious materials (SCMs) in cement is considered a promising bulk, sustainable, and harmless utilization method [2,3]. Cement building materials are required to follow low-carbon development guidelines, and the use of fine-grained tailings to prepare low-carbon mineral admixtures can improve the comprehensive utilization rate and reduce the production of Portland cement, thus reducing carbon emissions.

However, it should be noted that mineral tailings are not normally utilized in cement, and their use is accompanied by the reduced strength of cement and concrete mortars [4]. The activation and excitation of SCMs are essential prerequisites [5]. As an active SCM, the hydration product of ground quartz, feldspar, and mica is an amorphous C–S–H gel in the presence of calcium hydroxide [6,7,8]. The pozzolanic activity and hydration reaction properties have been explored, but the fluidity of blended cement and the consumption of PCE superplasticizers and water has not been discussed in the literature. Mechanical activation is a recommended method for activating mineral-processing tailings for its use in cement production, which can enhance the specific surface area of materials and accelerate the hydration rate through the production of new surfaces, phase transformation, and decrease in particle size [9]. However, tailing mineral admixture often suffers from poor flowability and PCE wastage [10,11,12]. A higher PCE dosage is needed to improve the fluidity, segregation tendency, and setting delays of a slurry, thereby enhancing the workability of mineral tailings admixture concrete [13]. This result was confirmed in a previous study by our group, which elucidated that mineral admixture prepared using copper-tailing-based blended cement reduced fluidity, wasted PCE, and increased cost [14].

Ince reported that beneficiation and activation processes refine tailings, leading to enhanced agglomeration, altered surface potential value, and increased water and admixture adsorption, and lead to the reduced working performance of SCM tailing concrete [10,11,12]. Most of the published research suggests that clay minerals in tailings cause low flowability. The reason is that clay can adsorb more water and reduce free water in slurry while increasing PCE adsorption by clay, which significantly diminishes the efficiency of PCE utilization [15,16]. The interaction between clay minerals and polycarboxylate superplasticizer can also be influenced by the pH value and the concentration of inorganic salt ions such as Mg^2+^ and Ca^2+^ [17]. The -COO- group on the PCE molecule forms complexes with the Ca^2+^ ions that are adsorbed onto the clay’s surface, leading to a decrease in the dispersed cement’s PCE content, subsequently diminishing mortar workability [18,19]. Nevertheless, previous studies have not examined the fluidity in activated minerals in order to analyze the reasons for reduced flowability, and it remains unclear how changes in mineral structure during activation processing affect SCMs’ abilities. Clay that has a large proportion of minerals (excluding other phases such as quartz, limestone, feldspars, etc.) exhibits higher pozzolanic activity, as well as increased water demand and PCE levels, as a consequence of its larger surface area [20]. It has been suggested that novel admixtures with improved effectiveness in highly negatively charged mineral cement blends need to be further developed [20]. Similarly, this decrease in workability imposes a constraint on the widespread adoption and application of mineral tailing admixtures. In most cases, the physicochemical properties of activated tailings significantly impact the paste’s fluidity and its compatibility with admixtures [21].

However, the main mineral components in tailings are usually not clays, and some tailings do not contain them at all. Therefore, these findings are not sufficient to help us solve the problem of SCM tailings’ performance optimization and application. The tailings primarily consist of quartz, feldspar, and mica, which are minerals characterized by their shelf and layered structures. However, so far, a systematic investigation into the impact of silicate mineral samples of different mineralogical composition on the performance of cement blends is still missing in the existing literature, especially regarding differences in the interaction with PCE superplasticizers and, in particular, with PCE adsorption.

This paper investigates quartz, feldspar, and muscovite mineral admixtures, elucidating the main factors affecting the slurry properties of mixed cement, which are crucial for optimizing the use of mineral admixtures in cementitious materials. The surface of silicate minerals is electronegative, and cement particles are positively charged. Clarifying the reaction and action mechanism of mineral admixture with cement, water, and other media under the effect of PCE modification can help us optimize the performance of mineral admixture cement paste and provide a theoretical basis for the development of functional admixtures to improve the performance, hydration process, and filling effect of mineral admixtures.

## 2. Materials and Methods

### 2.1. Silicate Minerals

**Figure 1:**
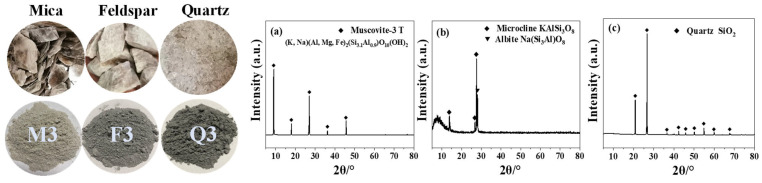
Morphology and XRD patterns of natural silicate minerals. (**a**) XRD patterns of mica (M), (**b**) XRD patterns of feldspar (F), and (**c**) XRD patterns of quartz (Q).

**Figure 2:**
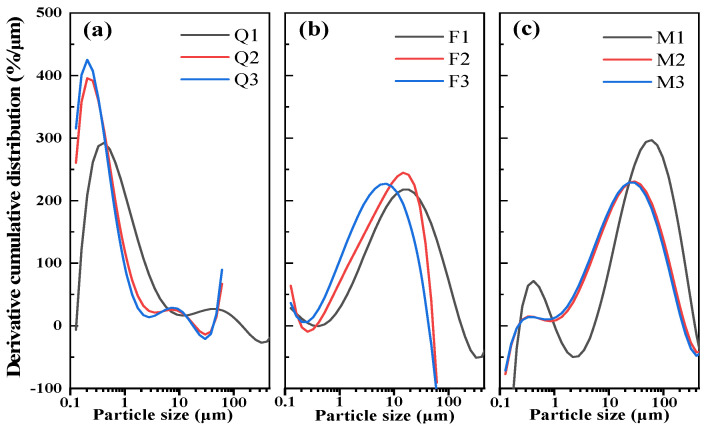
Derivative cumulative distribution of the silicate mineral admixture’s particle size. (**a**) Quartz admixtures, (**b**) feldspar admixtures, and (**c**) mica admixtures.

The silicate minerals used in this work are natural quartz (Q), feldspar (F), and mica (M) from Kunming, China; their morphologies, chemical components, and X-ray diffraction (XRD) patterns are given in Figure 1. The mechanical activation method was utilized for improving the particle size, crystalline structures, and surface properties of these minerals. A planetary ball mill, with a volume of 500 mL, was loaded with 500 g of the sample, along with 586 g of 20 mm stainless-steel spheres, 314 g of 15 mm stainless-steel spheres, and 183 g of 10 mm stainless-steel spheres. The speed of the mill was 400 rpm. Quartz, potassium feldspar, and mica were each ground by the XQM-12 planetary ball mills (Changsha Tianchuang powder Technology Co., Ltd., Changsha, China) to specific surface areas of 500 ± 50 m^2^/kg, 800 ± 50 m^2^/kg, and 1000 ± 50 m^2^/kg, named Q1, Q2, Q3; F1, F2, F3; and M1, M2, M3. The test method used to measure the specific surface area was the Blaine method, which was carried out according to the GB/T 8074-2008 standard [22], and the results are presented in Table 1. The particle size distribution (PSD) of the activated samples was measured using a BT-9300Z (Dandong Bettersize Instrument Co., Dandong, China) laser diffraction particle size analyzer. The particle size distributions are given in Figure 2.

### 2.2. Cement and PCE Superplasticizers

**Figure 3:**
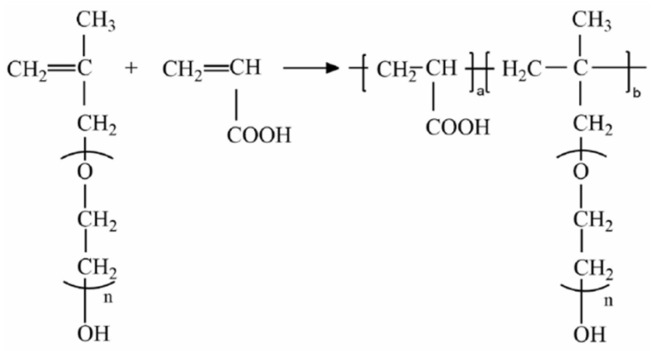
Chemical structure of the PCE used in the study.

Portland cement, supplied by the China Building Materials Research Institute, Beijing, China, meets the requirements of the GB 8076-2008 standard, Appendix A [23]. The PCE concrete additive, supplied by Jiangxi Dieter Technology Co., Ltd., Nanchang, China, has a solid content of 45% and is diluted at a 1:1 ratio with water. It is a precast type PCE, known as “HPEG”, a high-range water-reducing admixture, which provides long slump retention and is commonly used in ready-mix concrete to extend workability to over 2 h (pH = 4.31). The chemical structure is shown in Figure 3.

### 2.3. Chemical Reagents

Pure (99.9%) reagents (NaOH, KCl, AlCl_3_·6H_2_O, Ca(OH)_2_, and HCl) from Xilong Chemical Analytical (Shantou, China) were used. Solutions were prepared with distilled water and pure reagents: 0.1 mol/L NaOH, 0.1 mol/L HCl, 0.1 mol/L KCl, 0.02 mol/L AlCl_3_, and saturated Ca(OH)_2_. These were diluted 5 times, 10 times, and 20 times with 0.02 mol/L AlCl_3_ solution (where the Al^3+^ ion concentration was 540 mg/L) to obtain dilution solutions with Al^3+^ ion concentrations of 108 mg/L, 54 mg/L, and 27 mg/L. The solutions were then diluted 5 times, 6 times, 10 times, and 20 times with 0.1 mol/L KCl solution (the K^+^ ion concentration was 3900 mg/L) to obtain dilution solutions with K^+^ ion concentrations of 780 mg/L, 650 mg/L, 390 mg/L, and 195 mg/L.

### 2.4. Preparation

As shown in Table 2, two series of samples were prepared: one series without PCE and another series with PCE. The water-to-binder ratio (*w*/*b*) of the sample without PCE was 0.5, while the *w*/*b* of the sample with PCE was 0.34. The flowability of the PC pastes, both with and without PCE, was maintained at 100 ± 5 mm. The content of the silicate minerals was fixed at 30%.

The Al^3+^, K^+^, Ca^2+^, Fe^3+^, and Si^4+^ ion-leaching solutions from mineral particles under alkaline conditions were prepared as follows: Q3, F3, and M3 were mixed with 0.1 mol/L NaOH solution at a solid–liquid ratio of 3:1, stirred with a magnetic mixer for 3 min, and then left to settle for 2 min. The clarified solution was obtained by filtering with a nylon filter.

### 2.5. Experimental Methods

The particle size distribution (PSD) of the samples was measured using a BT-9300Z (Dandong Bettersize Instrument Co., Dandong, China) laser diffraction particle size analyzer. The information is presented as the specific surface area (SSA).

The fluidity of cement paste was determined using a 30 mm^⌀^ stainless-steel cylinder, in accordance with the Chinese standard GB/T 8077-2012 [24].

Rheological performance was tested using a RHEOMETER HAAKE MARS 60 (Themo Fisher, Waltham, MA, USA). The samples were stirred for 1 min, and then, the rheological properties were tested using a serrated rotor with a diameter of 20 mm. The test procedure involved the shear rate increasing from 0 to 500 S^−^ and then decreasing from 500 S^−^ down to 0. The rheological model was fitted with a one-way (at the time of descent) curve. The Herschel–Bulkley model was used to describe the rheological behavior of the cement paste.

A TOC (total organic carbon) analyzer was used to test the saturation adsorption of PCE. A 1 g amount of sample was added to 50 mL of PCE solution prepared with pure water at a concentration of 4 g/L. After stirring for 3 min and leaving it to stand for 4 min, the solution was filtered. Then, 1 mL of filtrate was taken and diluted 10 times to prepare the test sample. The amount of PCE adsorbed by the solid particles was determined by subtracting the total organic carbon content of the added 4 g/L PCE solution from the total organic carbon content in the filtrate.

The contact angle of the silicate minerals was measured using a Video Contact Angle Meter (JY-82C, Chengde, China). A 16 µL water droplet was used for the test, which was conducted using video recording with images captured every 50 ms.

At 25 °C, the zeta potential of the cement paste was determined using an A80030E zeta probe analyzer. The mineral samples were added to distilled water to form a 0.1‰ mineral solution. After stirring it for 2 min and leaving it to stand for 48 h, the solution’s zeta potential was determined at a given pH (using HCl, NaOH, and saturated Ca(OH)_2_ solutions) in the presence and absence of PCE at a concentration of 4 g/L. Then, the supernatant was quickly introduced into a sample beaker for the zeta potential test. Each sample was tested three times, and the average value was taken as the final result.

The surface chemistry of the activated mineral admixture was investigated using an X-ray Photoelectron Spectrometer (XPS Thermal Scientific K-Alpha, Thermo Fisher Scientific, Waltham, MA, USA), and the Advantage system was used for analysis. The X-ray source was Al Kα, operating at a voltage of 12 kV, with a step size of 0.1 eV.

An X-ray diffraction (XRD) test was performed on an X-ray diffractometer (DX-2700, Dandong, China) in the 2-theta range of 5–80° and 1–20° (step size, 0.02°; scanning speed, 5° per min and 1° per min) with the use of Cu Ka radiation (k = 0.15416 nm). The (001) layer spacing change after the mica’s PCE adsorption was calculated using the Bragg equation: 2dsinθ = n λ (λ = 0.15406 nm, n = 1).

The ICP–OES/MS (Agilent 7700s, Agilent Technologies, Inc., Santa Clara, California, USA) was used to test the content of dissolved metal ions in activated quartz, feldspar, and mica under alkaline conditions (pump rate, 20 r/min; nebulizer flow, 1.00 L/min; nitrogen gas, 1.00 L/min; sample flush time, 40 s; RF power, 1550 W).

## 3. Results and Discussion

### 3.1. Fluidity of the Cement Paste Blended with Silicate Minerals

**Figure 4:**
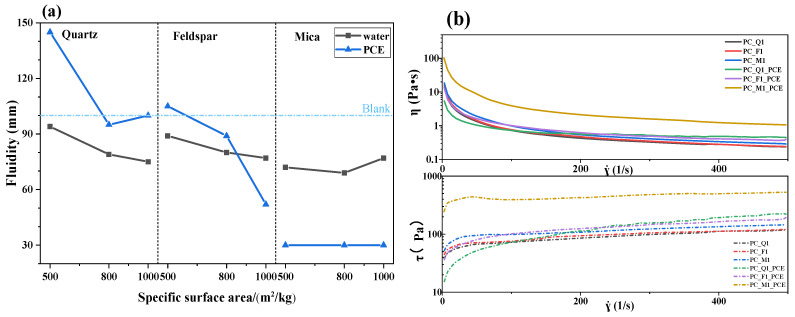
Fluidity (**a**) and viscosity (**b**) of the cement paste blended with silicate minerals.

The fluidity of the cement paste blended with silicate minerals is illustrated in Figure 4a. The blank represents the fluidity of the OPC paste. It is evident that the fluidity of the samples containing quartz and feldspar decreased as their specific surface areas increased. However, the fluidity of mica increased at the specific surface area of 960 m^2^/kg, which indicates that the refinement of the mica’s particle size is beneficial to the improvement of the fluidity of slurry-mixed cement. When the PCE was added, the fluidity of the sample containing quartz increased. Conversely, the sample containing feldspar initially increased and then decreased, while the sample containing mica completely lost its fluidity. This may be an effect of the different hydrophilicity of the minerals and the different adsorption with PCE.

The rheological properties of the paste of cement blended with silicate minerals are presented in Figure 4b, and the Herschel–Bulkley curve-fitting values are shown in Table 3. The viscosity of the samples follows the order M1 > F1 > Q1 or M1PCE > F1PCE > Q1PCE when PCE was added. The sample containing mica exhibited the highest viscosity, while the sample containing quartz had the lowest. With PCE addition, the τ_0_ decreased from 12.15 Pa to 3.12 Pa for the sample with quartz, decreased from 24.46 Pa to 11.84 Pa for the sample with feldspar, and increased from 30.36 Pa to 138.5 Pa for the sample with mica. These results indicate that the addition of PCE enhanced the viscosity of the sample containing mica while decreasing the viscosity of the samples containing quartz and feldspar. Therefore, minerals are the main factor affecting the rheological properties of the paste of cement. The differences in these minerals arise from the mineral crystal structure coordination, with the number of metal ions and aluminum ions coordinated. These differences may affect the hydrophilicity and PCE adsorption of minerals.

### 3.2. Wettability of Silicate Minerals

**Figure 5:**
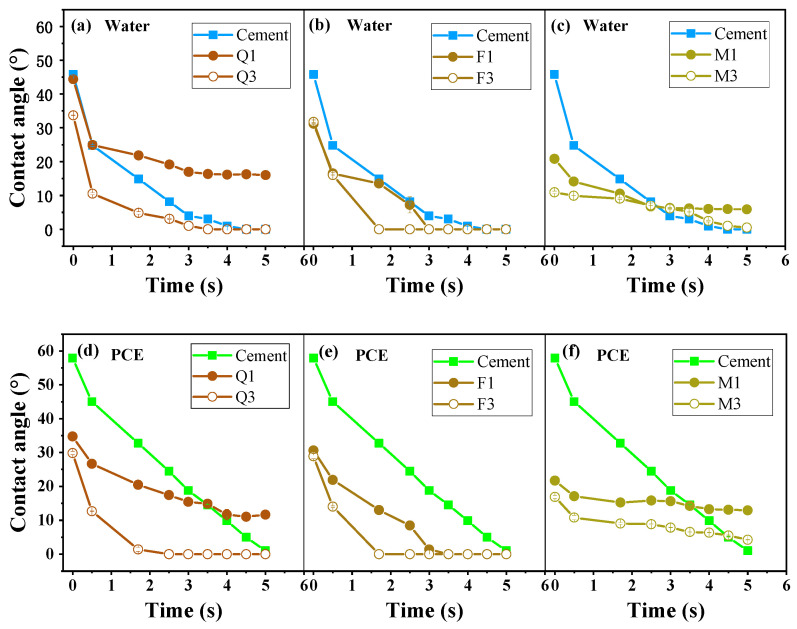
Wettability and dynamic contact angle of silicate mineral particles: (**a**) quartz in water, (**b**) feldspar in water, (**c**) mica in water, (**d**) quartz in PCE solution, (**e**) feldspar in PCE solution, and (**f**) mica in PCE solution.

The wettability of silicate minerals is shown in Figure 5. It can be seen that mica exhibits the highest hydrophilicity, while quartz has the lowest. Consequently, the paste of cement blended with quartz contains more free-flowing water. As the specific surface area increases, the contact angle of quartz and mica significantly decreases, while the contact angle of feldspar changes only minimally (Figure 5a–c). This indicates that the surface hydrophilicity of quartz and mica was enhanced through mechanical activation, while that of feldspar showed only minimal changes.

When the PCE was added, the fluidity of the cement paste blended with Q1 and F1 was higher than that of OPC, and the contact angles of Q1 and F1 were decreased (Figure 5d,e). This means that Q1 and F1 were more hydrophilic after the PCE was added, as it promoted the flow of free water in the paste, and, thus, the fluidity of the Q1- and F1-blended cement increased. The contact angles of M1 and M3 increased (Figure 5c,f). This means that M1 and M3 were less hydrophilic after the PCE was added, which is different from quartz and feldspar, and led to a decrease in fluidity. Thus, wettability is a factor in fluidity loss. As expected, mica is the most hydrophilic, resulting in increased viscosity, while quartz is the least hydrophilic, leading to the lowest viscosity and the best flowability.

The OPC displayed enhanced hydrophobicity after PCE adsorption, as evidenced by the change in the contact angle from 46° to 57°. In contrast, the mineral admixtures displayed enhanced hydrophilia with the addition of PCE. This reveals that the addition of PCE does not change the hydrophobicity of mineral admixtures and cause them to act as water-reducing agents, which is quite different from cement.

### 3.3. Adsorption of PCE on Silicate Minerals

**Figure 6:**
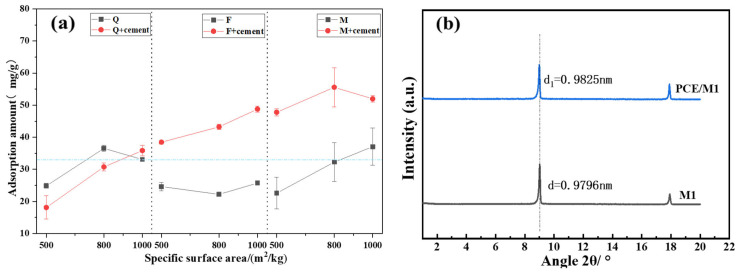
Adsorption of PCE on silicate minerals (**a**) and small-angle XRD of M1 (**b**).

Figure 6a depicts the adsorption amount of PCE. In general, silicate minerals adsorb less PCE compared to cement, as indicated by the blue dotted line. The adsorption levels of PCE increase after mechanical activation. Mica has the largest adsorption capacity of these silicate minerals, while quartz has the lowest.

Mica is a layered clay mineral, and such minerals cause PCE to undergo interlayer adsorption [19]. It has been reported that PCE molecules with long side chains are more likely to be inserted into the mica layer, leading to an increase in the adsorption amount [25]. Figure 6b presents the XRD small-angle diffraction results of mica particles that adsorbed PCE. The measured layer spacing increased by only 0.03–0.04 nm, which is significantly smaller than the size of the PCE side chains. Therefore, PCE cannot be adsorbed into the mica layer in an intercalated manner. This conclusion refutes some of the reported findings of mica’s intercalation adsorption [19,25].

### 3.4. Mechanistic Study

#### 3.4.1. Zeta Potential of Silicate Minerals

**Figure 7:**
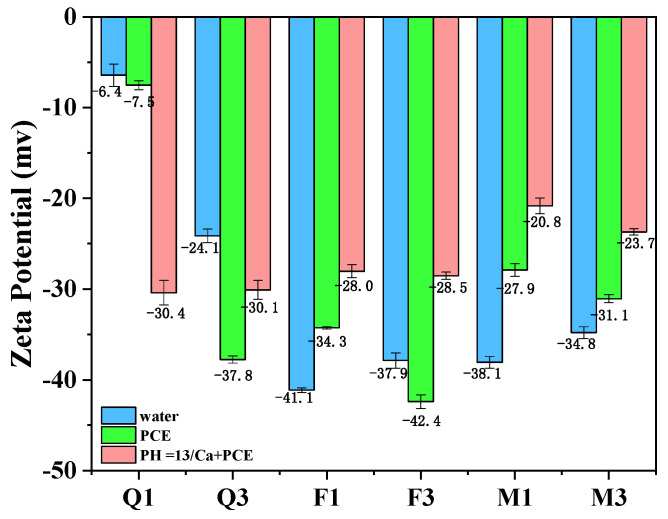
Zeta potential of different silicate mineral particles.

As shown in Figure 7, all the particles produced a negative surface charge. Q3 had a high value of −24.1 mV compared to Q1’s −6.4 mV. New surfaces were generated on the quartz after mechanical activation, and the unsaturated Si–O bonds interacted with water molecules to form hydrophilic hydroxyl groups, thus increasing the negative surface charge. The activated feldspar and mica also exhibited higher negative surface charges. When the PCE was added, the magnitude of the negative surface charge of Q3 increased from −24.1 mV to −37.8 mV, and that of F3 increased from −37.9 mV to −42.4 mV. PCE adsorption on silicate minerals results from van der Waals forces and electrostatic forces [18]. The broken silicon–oxygen bonds on the surface of silicate minerals have a strong polarizing effect. In the alkaline environment provided by the hydration of OPC, these bonds become negatively charged, adsorbing a large number of Ca^2+^ ions [26]. These results suggest that mechanical activation alters the surface structure and specific surface area of silicate minerals and enhances the adsorption of PCE on quartz, feldspar, and mica. At the same PCE dosage, excessive PCE levels molecules were produced in Q1- and F1-blended cements, which led to increased fluidity.

In the PCE solution with calcium hydroxide, the negative surface charge of Q1 increased to −30.4 mV, while the negative surface charge of other mineral admixtures decreased. This change can be primarily attributed to the presence of Ca^2+^. pH affects the electrical properties of mineral surfaces, and cations compress the electric double layer, reducing the surface potential of minerals [18]. As most silicate mineral admixtures exhibit a strong negative charge, Ca^2+^ is preferentially adsorbed on the negative surface, which is the same as in cement-based PCE solutions.

Therefore, it can be inferred that the adsorption of PCE on activated quartz is mainly due to hydrogen bonding, and PCE adsorption can be enhanced by Ca^2+^ complexation. The high negative surface charges of feldspar and mica admixtures result in electrostatic repulsion with PCE. Additionally, Ca^2+^ promotes the adsorption of PCE on activated mineral admixtures.

#### 3.4.2. Ionic Coordination of Silicate Mineral Admixtures

**Figure 8:**
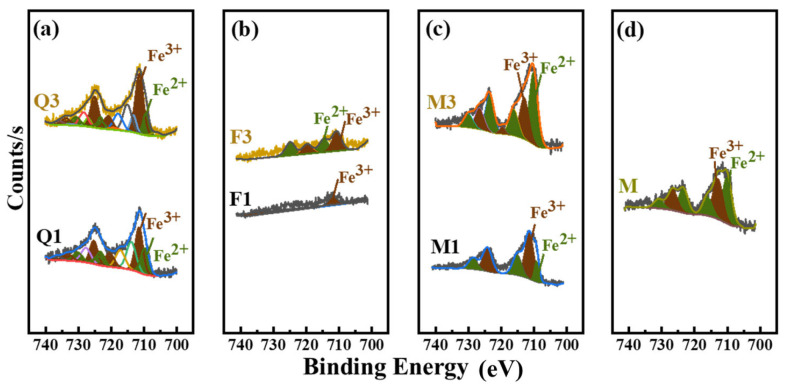
XPS spectra of silicate minerals. (**a**) XPS spectra of Q1 and Q3, (**b**) XPS spectra of F1 and F3, (**c**) XPS spectra of M1and M3, and (**d**) XPS spectra of M (M is the natural mineral mica).

Mechanical activation reduced grain size to the nanoscale, leading to an increase in lattice disorder [25]. The fragmentation of particles and the generation of new surfaces involve the rupture of chemical bonds [27]. Mica, a layered mineral, preferentially cleaves along the (101) interlayer under external forces, producing imbalanced coordination ions and high reactivity. Feldspar selectively breaks ionic coordination bonds, leading to an imbalance in coordination ions. The crystalline lattice of the quartz consists entirely of [SiO_4_]^4−^. Any fractures in this crystalline lattice result in the formation of at least two types of surface sites [28]. One is due to the homolytic cleavage of the ≡Si-O-chemical bond, which results in the generation of silyl ≡Si• and siloxyl ≡Si-O• radicals. The other, which is caused by the heterolytic cleavage of the ≡Si-O- chemical bond, allows the formation of the ≡Si^+^- and ≡Si-O^−^ charged species [29]. The depolymerization mechanism in feldspar and mica can be inferred as being caused by [(Al Si)_4_O_10_]. Free radicals and charged species are in a sub-stable state and tend to decay through direct recombination to form siloxane ≡Si-O-Si≡ units or through heterogeneous reactions with surrounding chemicals [30]. Thus, activated silicate minerals readily interact with water molecules to form ≡Si-OH and =Al-OH groups. Additionally, as can be seen in Figure 8a, Fe^3+^ or Fe^2+^ ions are easily introduced onto the surface of quartz particles during mechanical activation. As the activation time increases, the concentration of Fe^3+^ also increases. This phenomenon is attributed to the complexation between broken quartz and Fe^3+^ or Fe^2+^ ions facilitated through the grinding medium, resulting in the formation of Si-O-Fe bonds and reaching a state of equilibrium decay [31]. In Figure 8b, feldspar complexed poorly with Fe, indicating that feldspar does not easily complex with Fe in mechanically activated environments. In Figure 8c,d, M, M1, and M3 contain identical Fe^3+^ or Fe^2+^ ions, and Fe^3+^ or Fe^2+^ is mica’s interlayer ligand ion, making complexation with the medium difficult to analyze.

#### 3.4.3. Dissolved Metal Cation of Silicate Minerals

**Figure 9:**
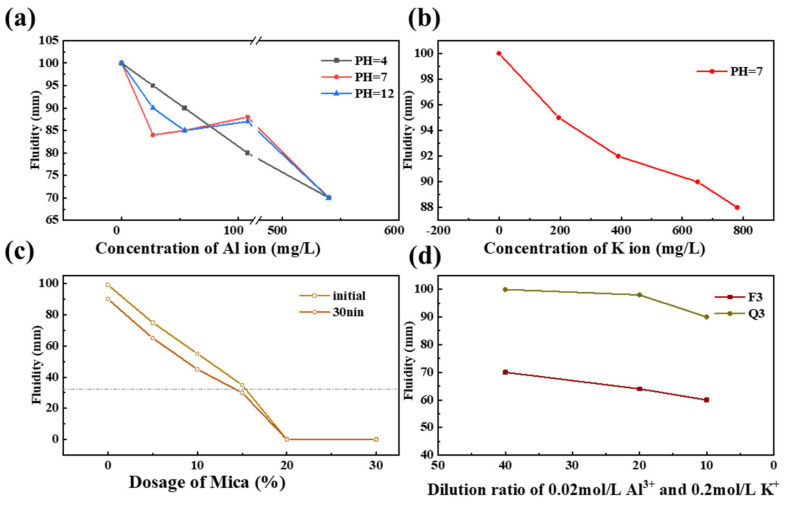
Fluidity with the additions of metal cations and mica: (**a**) cement paste fluidity with Al^3+^; (**b**) cement paste fluidity with K^+^; (**c**) mica SCM paste fluidity with differing mica levels; (**d**) quartz and feldspar paste fluidity with Al^3+^ and K^+^ concentrations.

The ion solubility of activated minerals was examined in alkaline solutions at a pH of 13 (Table 4). In Table 4, Mica exhibits the highest solubility of metal ions, and the solubility of Al^3+^ and K^+^ in feldspar and mica is tens to hundreds of times higher than that in quartz. Al^3+^ is expected to have a significant impact on PCE adsorption, leading to abrupt changes in the rheological properties of cement paste and accelerating the loss of flowability [32,33]. As shown in Figure 9a,b, Al^3+^ led to a reduction in the fluidity of paste. When the concentration of Al^3+^ reached 20 mg/L at a pH value of 7, the fluidity decreased by 17%, indicating that the formation of colloidal Al(OH)_3_ results in the loss of fluidity. The solubility of K^+^ from mineral particles was the highest, with a concentration of 280–400 mg/L, leading to a 5–8% decrease in fluidity. The dissolution of metal cations like Al^3+^ formed a colloidal structure, which increased viscosity and reduced fluidity. When there are an increased number of unsaturated Si–/Al–O bonds on mineral particle surfaces, ion solubility is caused by increasing activation [6,7]. Complexation and adsorption between PCE and dissolved cations have been identified as the main factors affecting the flowability of cement paste.

Mica exhibits the highest solubility of Al^3+^ and K^+^ ions. Figure 9c investigates the fluidity of cement paste containing various amounts of activated mica. As the level of activated mica increases, the fluidity decreases rapidly, eventually losing its fluidity when the amount reaches approximately 15%.

Figure 9d examines the fluidity of mineral-activated cement paste at different concentrations of metal cations. The tests revealed that, while metal cations adsorb PCE and reduce fluidity, it is the structure and surface properties of minerals that are primarily attributed as contributing to the fluidity of mineral SCMs.

#### 3.4.4. The Hydrolysis and Potential Equilibrium of Silicate Minerals

The development of acid groups on the surface of quartz and their dissolution are the sources of electrokinetic activity of quartz in aqueous solution, as shown in Equations (1)–(4). Zhang J et al. [34] proposed a mechanism for quartz charging in water involving (1) dissociation or adsorption under varying pH values, resulting in different surface electrical characteristics; (2) the adsorption of localized ions in aqueous solution, leading to hydroxyl surfaces; and (3) the breakdown of silica–oxygen bonds after the shattering of quartz crystals. Since dissociation and adsorption are reversible processes, quartz exhibits an overall negative electrical property over a wide pH range.

Dissolution equilibrium: SiO_2_ + 2H_2_O = H_4_SiO_4_ (1)

Lg [H_4_SiO_4_] = 0.151 − 1162/T (2)

Ionization equilibrium: H_4_SiO_4_ = H^+^ + H_3_SiO_4_^−^, pk = 9.8 (3)

−9.8 = −lg [H_4_SiO_4_] + lg[H^+^] + lg [H_3_SiO_4_^−^] (4)

**Figure 10:**
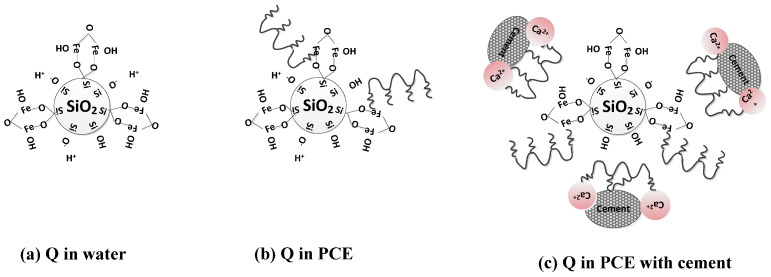
Schematic diagram of quartz hydrolysis and PCE adsorption: (**a**) the hydrolysis of quartz in water; (**b**) the hydrolysis of quartz in PCE solution; (**c**) the hydrolysis of quartz in PCE solution with cement.

According to Equation (2), the dissolution equilibrium of quartz is pH-independent, and H_3_SiO^4−^ is the dominant ion in the dissolved system. Consequently, in aqueous solutions, activated silicate minerals primarily adsorb PCE through hydrogen bonding. With increasing activation, the complexed Si-O-Fe on the surface of activated quartz enhances positive potential, leading to the increased hydrolysis of Fe and the generation of more hydroxyl ligands. The Fe–OH then adsorbs PCE to form a water complex adsorption, resulting in an increase in the amount of PCE adsorption. Upon adding cement, the solution becomes alkaline, and Ca^2+^, released from the hydration of cement, adsorbs PCE through ionic bonding. By combining these changes with the characteristics of surface potential on activated mineral particles, we can elucidate the mechanism behind the interaction between activated quartz and PCE, as shown in Figure 10.

After the activation of feldspar and mica, a significant number of Si-O and Al-O bonds are broken, exposing cations such as Na^+^, K^+^, and Ca^2+^ to the surface. This exposure enhances their solubility in water. Consequently, the mineral particles acquire a negative charge across the entire pH range [35]. The ionization of the silanol group (≡Si-O-H) and aluminol group (=Al-O-H) at the edge of the mica particles occurs with variations in pH. These changes in pH lead to deprotonation (or protonation) processes, resulting in variations in the ζ-potential and adsorption of PCE, as demonstrated by Equations (5)–(10) [36].

Dissolution equilibrium in aqueous solution: ≡Si OH ⇌ ≡Si O^−^ + H^+^ (5)

= Al-OH ⇌ = Al O^−^ + H^+^ (6)

Dissolution equilibrium in alkaline solutions: ≡Si OH + OH^−^ ⇌ ≡Si O^−^ + H_2_O (7)

=Al-OH + OH^−^ ⇌ = Al O^−^+ H_2_O (8)

Al^3+^ + 3OH^−^ ⇌ Al (OH)_3_ (9)

Al^3+^ + 4OH^−^ ⇌ Al (OH)_4_^−^ (10)

**Figure 11:**
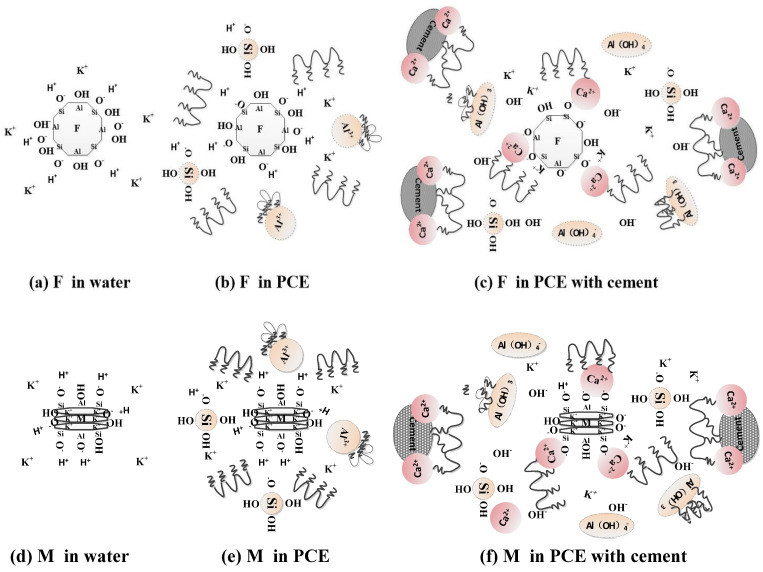
Schematic diagram of feldspar and mica hydrolysis and PCE adsorption: (**a**) the hydrolysis of feldspar in water; (**b**) the hydrolysis of feldspar in PCE solution; (**c**) the hydrolysis of feldspar in PCE solution with cement; (**d**) the hydrolysis of mica in water; (**e**) the hydrolysis of mica in PCE solution; (**f**) the hydrolysis of mica in PCE solution with cement.

The adsorption of metal elements such as Al^3+^ and K^+^ complexed with PCE, as well as the bridge-linkage adsorption of Ca^2+^ with PCE, are the primary forms of adsorption through activated feldspar and mica. In an aqueous solution, activated feldspar and mica exhibit a strong negative charge and electrostatic repulsion towards PCE. Mineral particles partially form hydrogen bonds with PCE and are also adsorbed through the formation of colloidal Al(OH)_3_ from dissolved Al^3+^ ions. When cement is added, the pH value increases, resulting in the deprotonation of the silanol group and aluminol group. This leads to an increased leaching rate for metal elements such as Al^3+^ and K^+^. Under alkaline conditions, Al^3+^ exists as Al (OH)^4−^, which then undergoes dehydration with -COOH groups on PCE to form a PCE-coated colloidal Al (OH)_3_. Due to its very low solubility product constant, aluminum hydroxide adsorbs PCE in its colloidal form, causing the condensation and coagulation of PCE. Additionally, the bridging effect of Ca^2+^ enhances ion-bonded adsorption between mineral particles on the surface with PCE. The process illustrating the adsorption of activated feldspar and mica particles with PCE is shown in Figure 11.

## 4. Conclusions

This study demonstrates that mechanical activation increases the hydrophilicity and water demand of mineral admixture. Additionally, activation promoted metal ion ligand chelation and potential changes. During mechanical activation, quartz forms Fe–O bonds with the grinding medium, leading to increased surface wettability. On the other hand, feldspar and mica possess cationic coordination structures, which make it challenging for coordination bonds to be formed with the grinding medium, thus causing minimal change in surface wettability. Capillary adsorption caused by particle refining is identified as the main reason for the water absorption of activated silicate minerals. Under alkaline conditions, cations from activated silicate minerals dissolve, increasing cement slurry viscosity and reducing PCE’s water-reducing impact significantly, resulting in substantial paste fluidity loss. The innovative conclusions reached are summarized as follows:

(1) Activated quartz hydrophilicity is similar to cement when it comes into contact with water. The water demand and PCE absorption are the lowest. Optimal flowability can be achieved at a specific surface area of 535 m^2^/kg. Increasing the specific surface area leads to coordination between minerals and Fe ions in grinding media, which leads to enhanced hydrophilicity and PCE adsorption.
(2) The capillary adsorption of activated feldspar results in a reduction in free water within the blended cement. Activated mica demonstrated strong hydrophilicity, a high metal cation solution, and a lamellar structure, which increased the shear stress and reduced flowability. Consequently, 15 wt% mica causes a complete loss of flowability.
(3) With cement hydration, the solubility of metal cations such as Al^3+^, K^+^, and Si^4+^ in activated mineral admixtures is facilitated, and the Ca^2+^ produced by hydration will be adsorbed on the mineral’s surface. This results in a significant reduction in cement slurry fluidity due to the increased levels of PCE.
(4) PCE can adsorb to the surface of cement particles to improve slurry properties but cannot adsorb directly to mineral admixtures. A specialized polymer additive for siliceous waste admixture, possibly with cationic groups, needs to be prepared to improve fluidity. The utilization of siliceous wastes will result in a meaningful contribution to environmental protection.

## Figures and Tables

**Table 1:** Mechanical activation of silicate minerals.

Sample	Mineral	Grinding Time (min)	Blaine Specific Surface Area (m^2^/kg)
Q1	Quartz	10	535
Q2		15	880
Q3		20	1016
F1	Feldspar	15	518
F2		20	785
F3		25	1014
M1	Mica	30	463
M2		45	833
M3		60	960

Large ball, 586 g, Φ = 2 mm; mid-sized ball, 314 g, Φ = 1.5 cm; small ball, 183 g, Φ = 1 cm.

**Table 2:** Mixture components of the samples investigated (g).

Sample	PC	Quartz	Feldspar	Mica	PCE	Water
PC (Blank)	500	0	0	0	0	250
PC_30Q	350	150	0	0	0	250
PC_30F	350	0	150	0	0	250
PC_30M	350	0	0	150	0	250
PC_PCE (Blank)	500	0	0	0	1.13	170
PC_30Q_PCE	350	150	0	0	1.13	170
PC_30F_PCE	350	0	150	0	1.13	170
PC_30M_PCE	350	0	0	150	1.13	170

**Table 3:** Herschel–Bulkley curve-fitting values of the cement paste blended with silicate minerals.

Sample	PC_Q1	PC_F1	PC_M1	PC_Q1_PCE	PC_F1_PCE	PC_M1_PCE
τ_0_ (Pa)	12.15	24.46	30.36	3.12	11.84	138.5

Herschel–Bulkley: τ = τ_0_ + Kη^n^.

**Table 4:** Solubility of activated mineral particles in the NaOH solution (mg/L).

	Al^3+^	Fe^3+^	K^+^	Li^+^	Ca^2+^	Si^4+^
Q3	0.16	0.5	8.66	-	0.21	13.17
F3	10.83	0.26	285.93	-	0.34	30.98
M3	23.92	0.1	437.78	2.86	0.07	24.53

## Data Availability

The raw data supporting the conclusions of this article will be made available by the authors on request.
